# Environmental contaminants of honeybee products in Uganda detected using LC-MS/MS and GC-ECD

**DOI:** 10.1371/journal.pone.0178546

**Published:** 2017-06-01

**Authors:** Deborah Ruth Amulen, Pieter Spanoghe, Michael Houbraken, Andrew Tamale, Dirk C. de Graaf, Paul Cross, Guy Smagghe

**Affiliations:** 1 Department of Crop Protection, Ghent University, Coupure Links, Ghent, Belgium; 2 Department of Livestock Industrial Resources, Makerere University, Kampala, Uganda; 3 Laboratory of Molecular Entomology and Bee Pathology, Ghent University, Krijgslaan 281 S2, Ghent, Belgium; 4 School of Environment, Natural Resources and Geography, Bangor University, Gwynedd, United Kingdom; Montana State University Bozeman, UNITED STATES

## Abstract

Pollinator services and the development of beekeeping as a poverty alleviating tool have gained considerable focus in recent years in sub-Saharan Africa. An improved understanding of the pervasive environmental extent of agro-chemical contaminants is critical to the success of beekeeping development and the production of clean hive products. This study developed and validated a multi-residue method for screening 36 pesticides in honeybees, honey and beeswax using LC-MS/MS and GC-ECD. Of the 36 screened pesticides, 20 were detected. The highest frequencies occurred in beeswax and in samples from apiaries located in the proximity of citrus and tobacco farms. Fungicides were the most prevalent chemical class. Detected insecticides included neonicotinoids, organophosphates, carbamates, organophosphorus, tetrazines and diacylhydrazines. All detected pesticide levels were below maximum residue limits (according to EU regulations) and the lethal doses known for honeybees. However, future risk assessment is needed to determine the health effects on the African genotype of honeybees by these pesticide classes and combinations of these. In conclusion, our data present a significant challenge to the burgeoning organic honey sector in Uganda, but to achieve this, there is an urgent need to regulate the contact routes of pesticides into the beehive products. Interestingly, the “zero” detection rate of pesticides in the Mid-Northern zone is a significant indicator of the large potential to promote Ugandan organic honey for the export market.

## Introduction

Agricultural production in African developing countries is expected to become increasingly reliant on pollinator services [1]. However, in response to the increasing challenge of providing food security in sub-Saharan Africa, farmers have been simultaneously encouraged to adopt intensive agricultural practises often characterised by widespread use of pesticides as foliar sprays and seed coatings [2]. Meaning service provision by bees is contingent upon their ability to effectively function in an increasingly challenging toxicological environment [3]. Recent studies documented on the exposure routes of agrochemicals on honeybee pollination services [4] with residues in nectar and pollen [5], surface water [6], floral secretions and plant exudates [7]. Honeybees store these products in the hive leading to exposure of brood, wax and honey to agrochemicals if present [8]. Multiple exposure routes to agrochemicals and their impact on colony health have been repeatedly shown to present synergistic interactions of chemicals and increased exposure of honeybees to other important stressors such as ectoparasitic mites (e.g. *Varroa destructor*), pathogens (e.g. *Nosema*) and food shortages [9–11].

The effective mitigation of the drivers of reduced pollination services in the developed world is challenging, whereas in developing regions such as sub-Saharan Africa these challenges may be substantially exacerbated due to the toxicological environment within which honeybees function. The relative functionality of managed bees can determine their productivity, which in turn has the potential to negatively affect those African households and businesses reliant upon bee products to sustain their well-being [12].

In Uganda, pesticide usage remains relatively unregulated and the use of counterfeited products inhibits monitoring as chemicals are frequently mislabelled [13]. The toxicological environment is compounded by the use of four classes of insecticides [organochlorines (DDT), pyrethroids, carbamates and organophosphates] to control disease vectors such as tsetse flies and *Anopheles funestus*, the highest prevalence of which occurs in Northern Uganda [14]. The tobacco and citrus agricultural systems of the region heavily rely on insecticide usage, particularly neonicotinoids [15]. Within this environmental context, many thousands of beekeepers, encouraged by global development organisations, are struggling to produce sufficient honey and wax to raise themselves out of poverty [12]. Studies investigating agrochemical contamination levels of Ugandan honeybee products are almost absent, although a Kenyan study reported the presence of fungicides (chlorothalonil), organophosphates (chlorpyrifos) and fluvalinate at low concentrations in pooled beeswax samples [16]. It is critical to the Ugandan apicultural sector to determine the levels of agrochemical contamination in hive products as Uganda is one of five African countries approved to export honey to Europe [17].

To ensure a high level of product quality control, a routine analytical pesticide monitoring is recommended [8], and such process requires highly sensitive and selective analytical methods [18]. Bees, honey and wax are challenging matrices to analyse due to the presence of interfering compounds, such as the complex array of compounds as chitin, lipids and protein in honeybees, and the pigments and lipids in honey [19], and the highly lipophilic compounds such as esters of long-chain aliphatic alcohols with fatty acids or hydroxyl-fatty acids, long chain hydrocarbons and trace levels of carotenoids in beeswax [19]. Failure of a clean-up process to eliminate these compounds affects the reliability of sample recovery, as well as impairing the analytical equipment through clogging [20]. Optimisation of the clean-up steps for lipids has previously used primary and secondary amines (PSA), C18 and graphitized carbon block (GCB) sorbents [21]. Limited application of the freezing step in the clean-up procedure has generally only been applied to beeswax [20,22]. However, honeybees and honey also contain such compounds. This study modified the multi-residue analysis for beeswax, honey and honeybees by incorporating a freezing step using an acetonitrile extraction solution, primary and secondary amines (PSA) and octadecyl (C18) sorbents to maximize elimination of lipids [21]. The use of a solid phase dispersion alone has been found to retain higher loads of matrix interference that tend to damage the chromatographic system after only a few injections [20]. A simpler version of the multi-residue method was necessary because of the need to adapt sample preparation methods to the listed agrochemicals to be analysed (Table 1) [22]. After validating the modified method, the residues of 36 pesticides were quantified in samples of honey, honeybees and beeswax as we collected from three agro-ecological zones of Uganda.

**Table 1:** List of analysed pesticides (alphabetical order), their chemical class, agricultural role and method validation results for both LC-MSMS and GC-ECD.

				Beeswax		Honeybees		Honey		LOD μg/kg	LOQ μg/kg
No.	Pesticide	Chemical Class	Role	%recovery	%RSD	%recovery	%RSD	%recovery	%RSD		
**1**	Acetamiprid	neonicotinoid	I	47.2	5.4	119.8	11.1	56.3	2.4	0.003	0.010
**2**	Azoxystrobin	strobilurin	F	85.5	8.8	136.3	8.7	58.0	13.0	0.003	0.010
**3**	Deltamethrin	pyrethroid	I	94.1	5.8	x	x	x	x	0.116	0.387
**4**	Boscalid	anilide	F	61.0	6.1	118.1	7.0	73.3	11.5	0.015	0.050
**5**	Carbendazim	carbamate	F	50.8	3.7	67.7	10.5	38.8	6.8	0.003	0.010
**6**	Carbofuran	carbamate	I	59.9	4.0	102.4	4.6	55.9	7.3	0.015	0.050
**7**	Chlorpyrifos	organophosphate	I	81.7	4.6	54.4	5.1	60.3	15.7	0.015	0.050
**8**	Clofentezine	tetrazine	I	63.8	3.6	80.1	6.9	78.3	16.6	0.030	0.100
**9**	Cyflufenamid	amide	F	48.2	2.5	61.3	7.9	68.0	15.7	0.015	0.050
**10**	Cypermethrin	pyrethroid	I	112.3	7.1	x	x	x	x	0.588	1.959
**11**	Cyprodinil	anilinopyrimidine	F	59.8	6.2	52.20	5.9	56.1	6.1	0.003	0.010
**12**	Difenoconazole	azole	F	94.7	3.3	62.3	10.2	77.6	14.0	0.003	0.010
**13**	Dimethoate	organophosphate	I	39.6	7.1	92.2	10.5	46.8	12.6	0.030	0.100
**14**	Endosulfan	organochlorine	I	82.2	9.7	x	x	x	x	0.055	0.184
**15**	Fenitrothion	organophosphate	I	61.3	6.4	366.9	66.0	80.7	17.4	0.150	0.500
**16**	Fenoxycarb	carbamate	I	71.9	2.5	89.6	9.4	67.8	12.6	0.003	0.010
**17**	Imidacloprid	neonicotinoid	I	55.2	15.9	111.2	19.3	50.9	8.2	0.030	0.100
**18**	Iprodione	dicarboximide	F	50.0	2.9	94.9	12.6	72.6	5.5	0.150	0.500
**19**	Malathion	organophosphorus	I	94.3	6.6	154.9	14.0	84.3	21.3	0.030	0.100
**20**	Metalaxyl	xylalanine	F	79.3	1.5	101.0	5.5	74.0	9.8	0.015	0.050
**21**	Methomyl	carbamate	I	110.0	5.6	111.5	13.3	133.1	4.5	0.018	0.059
**22**	O,P' -DDT	organochlorine	I	62.5	4.4	x	x	x	x	0.118	0.629
**23**	P,P' -DDD	organochlorine	I	74.4	4.4	x	x	x	x	0.056	0.187
**24**	P,P' -DDE	organochlorine	I	57.1	7.3	x	x	x	x	0.056	0.187
**25**	P,P' DDT	organochlorine	I	63.6	4.3	x	x	x	x	0.056	0.186
**26**	Penconazole	azole	F	82.1	7.9	99.7	4.0	73.6	12.3	0.003	0.010
**27**	Permethrin	pyrethroid	I	112.7	7.0	x	x	x	x	0.554	1.847
**28**	Piperonyl-butoxide	unclassified	I	61.7	4.5	96.5	4.5	83.0	22.4	0.030	0.100
**29**	Pirimicarb	carbamate	I	56.1	25.2	104.7	6.3	56.3	4.8	0.003	0.010
**30**	Profenofos	organophosphorus	I	56.6	3.3	70.2	8.9	70.6	13.4	0.003	0.010
**31**	Pyraclostrobin	strobilurin	F	67.2	3.1	93.0	3.4	72.7	17.9	0.015	0.050
**32**	Tebuconazole	azole	F	79.6	2.9	114.2	23.4	75.2	12.5	0.003	0.010
**33**	Tebufenozide	diacylhydrazine	I	72.1	13.9	118.1	24.8	90.3	12.4	0.030	0.100
**34**	Thiram	dimethyldithiocarbamate	F	60.3	7.9	53.6	0.7	98.5	29.8	0.030	0.100
**35**	Thiamethoxam	neonicotinoid	I	73.8	6.8	109.5	16.4	98.1	17.4	0.150	0.500
**36**	Trifloxystrobin	strobilurin	F	54.8	1.7	66.7	7.3	77.1	17.2	0.003	0.010

Symbol interpretation; x = hive product was not analysed; F = Fungicide and I = Insecticide

## Materials and methods

### Study area

The study was carried out in the primary honey-producing areas of Uganda [23,24] which included the West-Nile, Mid-Northern and Eastern agro-ecological zones of Northern Uganda.

Prior to the collection of samples for this study, ethical approval was obtained from the College of Veterinary Medicine, Animal Resources, and Biosecurity, Makerere University (No. SBLS.ADR.20. Ref 16).

### Site identification

Sample sites (apiaries) were purposively clustered (tobacco, citrus, control) based on agro-ecological zones and primary cropping systems. The cereal-cassava-tobacco production system, located in the West-Nile agro-ecological zone (Arua district) is the largest honey-producing zone of Uganda with a mean annual honey yield of 84,320 kg [25]. The common crops grown are finger millet, maize, sesame, cassava, sorghum, tobacco, soya beans, pigeon peas, cow peas and green grams. Tobacco is the major cash crop and a main source of livelihood for most the population in the district [26]. Due to the large tobacco farms, apiaries from this region were assumed to be potentially exposed to pesticide contamination as tobacco farming frequently entails intensive insecticide usage [27]. The citrus cropping system, located in the Eastern agro-ecological zone (Soroti district), produces a lower quantity of honey (16,310 kg/year) [25]. The main crops grown are finger millet, groundnuts, maize, cowpea, sunflower, cassava and sorghum [28]. Farmers in this ecological zone had been encouraged to place beehives in their citrus farms to increase crop yields. As fruit orchards are mostly affected by insect pests, it was assumed farmers would frequently and intensively apply pesticides potentially increasing contamination of hive products. Finally, the Mid-Northern agro-ecological zone (Kitgum district) was selected as the lack of developed intensive agricultural would be expected to correspond to a lower (or zero) risk of honey product contamination. The reported mean annual honey yield for this cluster is 27,500 kg [25]. The common crops grown are sesame (simsim), upland rice, green vegetables, beans, groundnuts, sorghum, millet cassava, pigeon peas and sunflower [29].

A list of individual beekeepers was obtained for each zone and individual apiaries were randomly selected. During random selection, each apiary was allocated a number, using the computer random number generator, and individual apiaries were then selected. In cases where the generated number was not in the list, the process was repeated until the required numbers for each region were obtained. For the study, only apiaries within a 3 km-range of a particular cropping system were considered as this is the maximum flight range for honeybees. Equal numbers of samples were collected in each of the three zones for honey bees (18), bees wax (5) and honey (8) during the dry season from October 2014 to February 2015 (S1 Table).

### Sample collection and preservation

For ease of inspection, only beehives with removable combs (Kenya top-bar and Langstroth hives) were included in the sample frame. A transparent polythene paper, supported by a wire, was held at the hive entrance for 5 min and approximately 30 honeybees were trapped to ensure that mainly foragers were collected. The adult honeybees were stored in an ice box for 1 h before being transferred into 15 ml-conical tubes with 10 ml of absolute ethanol (99% purity) (Sigma-Aldrich, Bornem, Belgium). A beeswax comb of 10 x 10 cm was cut from the brood chamber of each hive and stored in a plastic bag at 4°C, and honey was collected into 15 ml-conical flask bottles and stored at 4°C. Samples were then transported on dry ice to Ghent University (Belgium) for laboratory analysis.

### Laboratory analysis

This study adapted the QuECHERS method (Quick, Easy, Cheap, Effective, Rugged and Safe) commonly used in the multi-residue analysis of food matrices [5]. The procedure for honeybee, beeswax and honey sample preparation was adapted from [5].

The list of potential agro-chemicals to be screened was based on the list of agricultural pesticides authorised in Uganda by the Ministry of Agriculture, Animal Industry and Fisheries [30] and other products commonly used in tobacco, cassava, citrus and maize crops (all commonly grown in the study regions) (Table 1).

### Sample preparation and extraction

#### Honeybees

Four grams of honeybees were weighed into a 5 ml-screw cap tube containing 5 ml of acetonitrile. An air-cooling bullet blender was used to homogenise the honeybees at 12 rpm for 10 min. The content was transferred into a 50 ml-conical tube and another 5 ml of acetonitrile was added to make a total volume of 10 ml. The blended honeybee samples were agitated with acetonitrile for 3 min at 300 rpm. A mixture of disodium hydrogen sesquihydrate (0.33 g), trisodium citrate dehydrate (0.67 g), anhydrous magnesium sulphate (2.66 g) and sodium chloride (0.67 g) was added to this extract in a 50 ml-conical tube to remove lipids, sterols and other compounds. The content of the salts was shaken at 300 rpm for 3 min to mix the salts with the honeybee sample. The samples were frozen overnight at -80°C before centrifuging at 10,000 rpm for 5 min. From this content, 1 ml of the supernatant was collected into a 10 ml-tube and 9 ml of Milli Q water was added to obtain 10 ml. Finally, the purified extracts were analysed using LC-MSMS and GC-ECD [31].

#### Beeswax

For the beeswax, samples were first blended and 2 g placed into a 50 ml-conical tube before adding 10 ml of acetonitrile. Samples were placed in a water bath at 80°C for 40 min to melt the beeswax. The contents were shaken for 15 s and placed back in the water bath. This was repeated four times to ensure complete melting of the beeswax. Extracts were frozen overnight at -80°C. Frozen extracts were centrifuged at 10,000 rpm for 5 min to separate the beeswax from the acetonitrile solution. Finally, 7 ml of the supernatant was pipetted into a 15 ml-dispersive solid phase extraction tube (d-SPE tube) packed with primary secondary amines (PSA) and octadecyl (C18) to remove any organic acids, polar pigments and other compounds that could interfere with the analysis. The contents were then shaken for 1 min and centrifuged at 3000 rpm for 5 min to obtain 1 ml of purified extract of the sample. The extracted samples were stored in glass screw caps bottles for LC-MSMS and GC-ECD analyses [31].

#### Honey

Five grams of honey sample was placed into 50 ml-conical flasks before adding 10 ml of water. Samples were placed in a water bath for 15 min at 60°C to dissolve the honey. Afterwards, 10 ml of acetonitrile was added and samples were shaken for 1 min. The sample was subsequently cleaned using a mixture of disodium hydrogen sesquihydrate (0.5 g), trisodium citrate dehydrate (1 g), anhydrous magnesium sulphate (4 g) and sodium chloride (1 g). The content was mixed by shaking at 300 rpm for 10 min. The samples were frozen overnight at -80 ^o^C before centrifuging at 6000 rpm for 5 min at 4°C. Then 7 ml of the supernatant was collected. Each sample was diluted 10 times by adding 1 ml of the sample to 9 ml of Milli Q water before LC-MSMS and GC-ECD analysis [31].

#### Reagent and apparatus

Analytical grade reagents of 99% purity were used in the experiments. Compounds as acetonitrile, anhydrous magnesium sulphate, disodium hydrogen sesquihydrate, trisodium citrate dehydrate and sodium chloride, as well as 50 ml-polypropylene centrifuge tubes, 15 ml-dispersive solid phase extraction tubes (d-SPE) packed with primary secondary amines and octadecyl, were obtained from Thermo-Fisher Scientific (Dreieich, Germany). The salts and all pesticide standards were obtained from Sigma-Aldrich.

#### Liquid chromatography mass spectrometry (LC-MS/MS)

All analyses were performed on an ACQUITY UPLC of Waters (Zellik, Belgium), equipped with a quaternary pump and membrane degasser. The separation column was kept at 40°C. An automatic injector was set to inject 10 μl per sample. The mobile phase components were (A) a 10 mM ammonium acetate solution in water and (B) acetonitrile with 0.1% formic acid. The gradient used was initially set at a flow rate of 0.4 ml/min of 98% mobile phase A for 0.25 min. From 0.25 min to 7 min, a linear gradient was used to 98% mobile phase B, which was maintained for 1 min. Then, a linear gradient was used to 98% mobile phase A and maintained for 1 min.

Sample analyses were performed using a triple quadrupole system with ESI (ACQUITY UPLC, xevo TQD mass spectrometer; Waters). More information on the equipment and technical settings used is provided by [31].

All analyses were performed with electrospray ionisation in positive ion mode. The capillary needle was maintained at +2 kV. For operation in the MS/MS mode, the following parameters were set: curtain gas (N_2_) at 7 bar; temperature 500°C. The analytes were monitored and quantified using MRM. Optimization of the MS/MS conditions, identification of the parent and product ions, as well as the selection of the cone and collision voltages, were performed with direct infusion of their individual standard solutions. Every individual standard pesticide solution was prepared in the concentration of 1 mg/ml in water/acetonitrile. The Masslynx software was used for the LC-MS/MS system control and data analysis. After the optimization of the collision cell energy of the triple quadrupole, two different m/z transitions were selected for each analyte, one for quantification (QIT) and one for confirmation (CIT) [31].

#### Gas liquid chromatography with electron capture (GC-ECD)

Halogenated compounds such as P,P’ DDE, P,P’ DDD, O,P’ DDT, P,P’ DDT, permethrin, cypermethrin, endosulfan and deltamethrin were screened using an Agilent technologies 6890N GC-ECD with an auto-sampler as described by [32].

Separation was performed on a HP-5MS (5% phenyl methyl siloxane) capillary column (30 m x 0.25 mm x 0.25 μm film thickness). The operating conditions were as follows: the column was initially set at a temperature of 80°C, then increased at a rate of 30°C/min to 205°C and held for 4 min. It was further increased at a rate of 20°C/min to 290°C and held constant for 8 min, followed by an increase at a rate of 50°C/min to 325°C. The temperature of the injector and detector were maintained at 280°C and 300°C, respectively. Helium was used as a carrier gas at a flow rate of 1.1 ml/min and the injections were made in the split mode with a split ratio of 52.7:1 [31].

### Method validation

Several attributes of the extraction method were validated: accuracy (percentage recovery), precision (%RSD), limit of detection (LOD), limit of quantification (LOQ) and linearity. The validation process involved six replicates of spiked samples prepared with known concentrations of pesticides (Table 1). As a proxy measure for method validation, we used a bee-representative matrix with workers of bumblebees (*Bombus terrestris*) that are known to be free of pesticides and these bees were obtained from a commercial producer (Biobest, Westerlo, Belgium). For the honey and beeswax samples, the samples were first evaluated and only samples free from pesticides were used as blank reference material. Then 10 μl of the pesticide standard solution (10 mg/l) was spiked into the blank samples. The spiked samples were left for 1 h to allow sample absorption of the pesticide before being subjected to the extraction and clean-up process. From the spiked samples, the LOD together with the LOQ were calculated by multiplying the standard deviation of the detected pesticide concentrations from the replicates by 3 and 10, respectively [32]. The percentage recovery and percentage relative standard deviation (RSD) were calculated to determine the method’s accuracy and precision, respectively. The percentage recovery was calculated by dividing the recovered concentrations by spiked concentration, and the % RSD was obtained by dividing the standard deviation by the average concentration [32]. To determine linearity, five different concentrations of the stock solution (0.1, 0.05, 0.01, 0.005, 0.001 mg/l) were prepared by dilution with acetonitrile/water (10/90) to form a calibration curve.

### Data analysis

The descriptive statistics were generated to capture frequencies of occurrence and distribution of individual pesticides across the analysed hive matrix products and apiary locations.

## Results

### Method validation

The validation of the multi-residue method showed that in the honeybee and honey samples, a respective number of 18 and 16 pesticides had recoveries within the acceptable range of 70–120%, while in the beeswax samples 15 pesticides had recoveries within the acceptable range. The precision was reliable as most % RSD were <20 and linearity coefficients for all compounds were within an acceptable range (r^2^>0.995). The LOQ for the LC-MS/MS ranged between 0.028 μg/kg and 0.094 μg/kg, while for GC-ECD the LOQ ranged between 0.200 μg/kg and 0.696 μg/kg. This method appears to be suitable for detecting most of the listed compounds (Table 1).

### Status and distribution of pesticide contamination

Of the 93 samples analysed for pesticides, 17 contained residues. All samples of beeswax collected from the Eastern and West-Nile agro-ecological zones revealed the presence of pesticides. Approximately 10 pesticides per beeswax sample were detected from these two regions. No pesticides were identified in beeswax collected from the Mid-Northern agro-ecological zone. Fourteen samples of honeybees out of the 54 analysed contained pesticides. Sixty percent of these originated from apiaries sited near citrus farms (Eastern agro-ecological zone), while the remaining 40% were from areas adjoining tobacco farms. No pesticide was detected in honeybee samples from the Mid-Northern agro-ecological zone. No pesticides were detected in honey in any of the zones.

Of the 36 pesticides analysed, 20 were primarily detected in honeybees and beeswax samples. Twelve of the 20 detected pesticides were insecticides and eight fungicides. The chemical classes of the 12 detected insecticides included neonicotinoids (4), organophosphates (3), carbamates (2), organophosphates (1), tetrazines (1) and diacylhydrazines (1). The most frequently detected compounds were cyprodinil, fenoxycarb, fenitrothion, carbendazim, tebuconazole and iprodione, whilst thiamethoxam, dimethoate, thiram, clofentezine and imidacloprid were the least frequently detected. All the above-mentioned pesticides were found in beeswax, with methomyl and cyprodinil being present in the honeybee samples. Citrus farms (Eastern zone) had the highest number of detected pesticides, followed by tobacco farms and the other agricultural cropping areas (Table 2). The Eastern (citrus farms) and West-Nile (tobacco farms) ecological zones had the highest incidence of contaminated beehive products compared to the Mid-Northern (other farms) ecological zone (Tables 2 and 3).

**Table 2:** Number of positive samples on the number of samples analysed (n) with detected pesticides over the different hive products (beeswax, bees, honey) and agro-ecological zones.

Pesticide	Class	% positive samples (n = 93)	Beeswax (n = 15)	Bees (n = 18)	Honey (n = 8)	Eastern zone (n = 31)	West-Nile zone (n = 31)	Mid-Northern zone (n = 31)
Methomyl	I	6.45	x	6	x	6	x	x
**Carbendazim**	F	13.97	13	x	x	7	6	x
**Acetamiprid**	I	6.45	6	x	x	4	2	x
**Cyprodinil**	F	25.81	12	12	x	15	10	x
**Dimethoate**	I	2.15	2	x	x	2	x	x
**Thiram**	F	3.23	3	x	x	1	2	x
**Thiacloprid**	I	10.75	10	x	x	6	4	x
**Imidacloprid**	I	4.30	4	x	x	2	2	x
**Fenitrothion**	I	15.05	14	x	x	9	5	x
**Metalaxyl**	F	2.15	2	x	x	2	x	x
**Penconazole**	F	8.60	8	x	x	5	3	x
**Thiamethoxam**	I	1.08	1	x	x	1	x	x
**Fenoxycarb**	I	16.13	15	x	x	8	7	x
**Clofentezine**	I	2.15	2	x	x	2	x	x
**Tebuconazole**	F	13.98	13	x	x	8	5	x
**Iprodione**	F	12.90	12	x	x	8	4	x
**Chlorpyrifos**	I	10.75	10	x	x	6	4	x
**Tebufenozide**	I	7.52	7	x	x	3	4	x
**Profenofos**	I	12.90	12	x	x	8	5	x
**Trifloxystrobin**	F	8.60	8	x	x	5	3	x

Key F = fungicides; I = insecticides; x = pesticide not detected; No. = number; % = percentage

**Table 3:** Mean residue levels of detected pesticide residue concentrations in the samples of beeswax and honeybees, the maximum residue limits (MRL, according to EU), the 48 h-lethal concentrations in honeybees by contact and oral exposure, and the types of pesticide-dependant crops observed around the apiary.

No	Pesticide	Beeswax (mean±SD) (μg/kg)	Honeybees (mean±SD) (μg/kg)	MRL (EU)	Contact acute 48 h-LD_50_ (μg/bee)	Oral acute 48 h-LD_50_ (μg/bee)	Pesticide-dependant crops in study area
1	Acetamiprid	0.012 ±0.021	x	0.05	8.09	14.53	Cotton, fruits, vegetables
**2**	Carbendazim	0.006 ±0.003	x	1	> 50	> 756	Beans, cereal, chickpeas
**3**	Chlorpyrifos	0.052 ±0.028	x	0.05	0.059	0.25	Cotton, fruits, grains
**4**	Clofentezine	0.037 ±0.004	x	0.05	> 84.5	> 252.6	Fruits
**5**	Cyprodinil	0.023 ±0.012	0.005 ±0.002	0.05	> 784	112.5	Fruits
**6**	Dimethoate	0.146 ±0.139	x	NA	0.12	NR	Cotton, fruits (citrus), sorghum, soybean
**7**	Fenitrothion	0.877 ±0.579	x	0.01	0.16	0.2	Cereals, cotton, fruits, vegetables
**8**	Fenoxycarb	0.005 ±0.001	x	0.05	> 204	> 204	Cotton, fruits
**9**	Imidacloprid	0.044 ±0.023	x	0.05	0.081	0.0037	Cereals, maize, rice, tobacco
**10**	Iprodione	0.218 ±0.008	x	0.05	> 200	> 25	Cotton, fruits, sunflower, vegetables
**11**	Metalaxyl	0.022 ±0.002	x	0.05	200	269	Citrus, cotton, onions, soybean, tobacco, tomatoes
**12**	Methomyl	x	0.061 ±0.051	0.02	0.16	0.28	Cotton, fruits
**13**	Penconazole	0.006 ±0.005	x	0.05	> 30	> 112	Cotton, tomatoes, vegetables
**14**	Profenofos	0.019 ±0.005	x	0.05	0.095	NR	Cotton, maize, potatoes soybean, tobacco
**15**	Tebuconazole	0.004 ±0.001	x	0.05	> 200	> 83.05	Cereals, fruits peanuts, peas, vegetables
**16**	Tebufenozide	0.040 ±0.008	x	0.05	> 234	> 100	Fruits
**17**	Thiacloprid	0.046 ±0.070	x	0.2	38.82	17.32	Cabbage, citrus, peas, potatoes,
**18**	Thiamethoxam	0.210 ±0.000	x	0.05	0.024	0.005	Citrus
**19**	Thiram	0.102 ±0.114	x	NA	> 100	> 106.8	Beans, cereals, fruits, millet, peas, sunflower, tomatoes
**20**	Trifloxystrobin	0.006 ±0.003	x	0.05	> 200	> 200	Cereals, fruits, vegetables,

Key. NA = data was not available, x = pesticide not detected, NR = information was not reported in the pesticide properties database used. The toxicological properties reported in the above table were collected from [34]

### Concentration levels (μg/kg) of detected pesticides

Most of the detected pesticides were traces, and only eight were detected above the LOQ [acetaprimid, cypridinil, dimethoate, thiram, thiacloprid, fenitrothion, chlorpyrifos and profenofos (comprising six insecticides and two fungicides)]. Of the six detected insecticides with a residue >LOQ, two were neonicotinoids, three were organophosphates with one organophosphorus. The majority of the detections >LOQ was from apiaries in the forage vicinity of citrus farms (Eastern zone), followed by tobacco farms (West-Nile zone). The Mid-Northern zone can be scored as “zero” for pesticide residues. All detections >LOQ were found in beeswax.

The highest mean concentrations found were for fenitrothion, thiacloprid, thiamethoxam, dimethoate, iprodione, thiram and chlorpyrifos, ranging from 0.052 to 0.877 μg/kg. The lowest mean concentrations were for trifloxystrobin, tebuconazole, penconazole, acetamiprid and carbendazim, ranging from 0.004 to 0.012 μg/kg. No maximum residue limits (MRLs) are defined for beeswax or honeybees. However, MRL values exist for honey and other apiculture products [33] and these were used as reference values in this study. All detected pesticide concentration levels were below the MRLs set by the European Union (Table 3). In this table, the μg/kg-residue levels detected are compared to μg/bee-lethal doses to help in contextualising the magnitude of threat to the honeybee’s health. However, as most residues were detected in the beeswax, this risk was perceived as very low.

## Discussion

This study developed a validated, multi-residue method for analysing 36 pesticides through the inclusion of an overnight-freezing step which minimized interference from wax particles such as fats. The under-pinning principle of incorporating a freezing out-step in the process lies in the differential melting points of lipids and organic solvents such as acetonitrile. Indeed acetonitrile melts at approximately -45°C, while most lipids melt at room temperature. Based on this difference, the frozen fraction with the lipids can be properly separated during the centrifugation step from the liquid fraction of the acetonitrile containing the pesticides/agrochemicals [35]. Interestingly, most of the pesticide residues detected in this study had not previously been recorded in East African [16] and Ugandan beeswax.

No pesticides were detected in honey, implying that Ugandan honey from this region is currently safe as per EU guidelines [33]. Beeswax was the most contaminated hive product, while only few traces of fungicides on honeybees. Contamination of beeswax occurs when honeybees collect contaminated nectar, water or pollen from their surrounding environment [5] Consequently, an increased likelihood of wax contamination arises as it is used to store all hive contents (honey and pollen).

Most of the pesticide residues detected in this study can be linked to agricultural activity. Citrus- and tobacco-dominated farms had higher residues compared to the less intensive agricultural cropping areas of the Mid-Northern region. Beeswax is not generally recycled for use by beekeepers in Uganda as most comb in the hive is harvested with the honey each season, approximately once every four months. Therefore, we expect that most of the detected pesticides is collected and/or brought back to the hive as a consequence of recent (preceding four months) rather than longer-term historical exposure. Citrus plants are commonly attacked by pests such as aphids requiring farmers to apply agrochemicals such as thiacloprid, thiamethoxam, dimethoate etc.[36]. Similarly, tobacco is a monocrop and vulnerable to a variety of pests and diseases requiring large amounts of pesticides [27]. Compounds such as chlorpyrifos and imidacloprid are commonly used [15,27]. Some of the agrochemicals are applied in all growth stages of the plants including the flowering period when honeybees tend to visit the plants for nectar and pollen. Moreover, indiscriminate application of sometimes unlabelled pesticides has been noted in the study areas [13]. Furthermore, apart from tobacco and citrus, exposure to agrochemicals also rises from other crops cited to be visited by honeybees such as soybean, cowpeas, maize and beans [37,38].

Some of the detected compounds are known to be highly toxic to honeybees (chlorpyrifos, dimethoate, fenitrothion, imidacloprid, methomyl, profenofos, thiamethoxam, thiacloprid and acetamiprid) [34], and two of these are prohibited to be used in seed treatment of melliferous crops (imidacloprid and thiamethoxam) [39]. However, no similar ban exists for the United States of America and Asia from where Uganda sources many of its plant protection products. Ugandan agriculture is increasingly turning to the use of insecticide-coated seeds in crops such as beans, cow peas and maize in an attempt to mitigate food security concerns [40]. Neonicotinoids are the most common compounds used in such seed coatings [41]. Once introduced to the environment, neonicotinoids are known to persist. Due to their high water solubility they are easily translocated from the soil into the plants where they accumulate in plant tissue, pollen and water secretions [41,42]. These compounds are also applied as foliar treatments in citrus and tobacco plants [42]. Based on solubility, one would expect that honey and honeybees contain more neonicotinoids than beeswax. However, our study finds the opposite. One possible explanation is that there could have been remnants of honey and pollen in the beeswax, since beeswax is the store for most hive products. Furthermore, samples were collected during the honey-harvesting season, and therefore we cannot rule out the presence of honey and pollen in the beeswax samples.

Of increasing concern for the scientific and apicultural sector are the synergistic effects of neonicotinoids and fungicides [11,43]. Even at very low concentrations (μg/kg), a combination of neonicotinoids (e.g. thiamethoxam) and fungicides (e.g. tebuconazole) has been found to increase neonicotinoid toxicity [43], suggesting a relationship between fungicide and increased neonicotinoid toxicity. Also, imidacloprid has been associated with impaired olfactory behaviour in honeybees [44], increased stress levels in adult bees leading to both lower foraging activity and colony growth [11], and causing immunosuppression which adversely affects honeybee antiviral defence mechanisms [4]. Honeybee sensitivity to insecticides varies across genotypes and age-classes [11,45] and appears to be further aggravated following neonicotinoid exposure when compared to exposure of other chemical classes such as organophosphates and pyrethroids and also fungicides [45]. Determining how Ugandan honeybee genotypes respond to exposure of neonicotinoids, fungicides and other pesticides or chemical contaminants will be important to our understanding of honeybee resilience in contaminated environments.

Whilst honey production in the study regions was within the EU limits for MRLs [33] and can therefore be considered safe for human consumption, the detection of unauthorised pesticides in the beeswax [39] presents challenges to the apicultural sector both in terms of meeting the EU standards and honey bee management. Honey-based products such as cut and chunk comb (i.e. sections of honey comb are incorporated into jars of honey) also have the potential to increase primary human exposure routes to harmful compounds [46]. These challenges are particularly acute in respect to beeswax production as they increase human exposure through secondary routes such as consumption of food additives, coating agents in pastry preparation, capsules and tablets [47].

Both honey and beeswax from developing countries have previously been sought due to the comparatively lower residue levels [48]. The detection of pesticides, even as traces, in Ugandan hive products presents a significant challenge to the burgeoning organic honey sector. But to achieve this, there is an urgent need to regulate the entry routes of these pesticides into the beehive products. Finally, the “zero” detection rate of pesticides in the honey samples from the Mid-Northern zone is a significant indicator of the large potential to promote the export market for Ugandan organic honey.

## Conclusion

This study developed a validated multi-residue method for pesticides/agrochemicals detection in honeybee hive products, using GC-ECD and LC-MSMS which simultaneously controlled for 36 pesticides. Various traces of insecticides and fungicides in bees and bee products were found from three agro-ecological zones in Uganda (West-Nile, Mid-Northern, Eastern), and the common chemical classes of insecticides detected included neonicotinoids, carbamates, tetrazines and diacylhydrazines. Almost all detected chemicals were found in the beeswax and in the samples derived from neighbouring citrus (Eastern) and tobacco (West-Nile) cropping systems, but the detected levels were below the MRL set by the EU as well as the lethal acute doses for honeybees. The presence of neonicotinoids and systemic fungicides suggests that whilst MRLs are low, possible sub-lethal impacts of several substances may present a threat to honeybees in the region. This suggests the need for a robust risk assessment of pesticides or chemical contaminants for our understanding of the resilience of Ugandan honeybee genotypes in a contaminated environment. Interestingly, the “zero” detection rate of pesticides in the honey from the Mid-Northern zone indicates substantial potential for marketing Ugandan organic honey in international markets.

## Supporting information

S1 Table List of samples and GPS coordinates. (DOCX)
