# Supplementary material for: Environmental contaminants of honeybee products in Uganda detected using LC-MS/MS and GC-ECD
Source: PLoS One. 2017 Jun 1;12(6):e0178546. doi: 10.1371/journal.pone.0178546 (PMC5453540; doi:10.1371/journal.pone.0178546)
Supplement: S1 Table — (DOCX) [file pone.0178546.s001.docx]

Table 1: List of samples and GPS coordinates

| No | Sample code | Northing | Easting | Elevation | Honey (n=24) | Beeswax (n =15) | Bees (n= 54) |
| --- | --- | --- | --- | --- | --- | --- | --- |
| 1 | K1 | 3.33649 | 32.9305 | 976 | √ | √ | √ |
| 2 | K2 | 3.27662 | 32.86525 | 927 |  |  | √ |
| 3 | K3 | 3.23672 | 32.94607 | 1001 | √ | √ | √ |
| 4 | K4 | 3.25804 | 32.94335 | 962 |  |  | √ |
| 5 | K5 | 3.32541 | 32.94785 | 948 | √ |  | √ |
| 6 | K6 | 3.31999 | 32.92083 | 961 | √ | √ | √ |
| 7 | K7 | 3.26883 | 32.94841 | 969 |  |  | √ |
| 8 | K8 | 3.31118 | 32.94246 | 941 | √ | √ | √ |
| 9 | K9 | 3.31783 | 32.92669 | 949 | √ |  | √ |
| 10 | K10 | 3.27121 | 32.94735 | 980 | √ | √ | √ |
| 11 | K14 | 3.35818 | 32.68202 | 1002 |  |  | √ |
| 12 | K8B | 3.31118 | 32.94246 | 941 |  |  | √ |
| 13 | K9A | 3.31783 | 32.92669 | 949 | √ |  | √ |
| 14 | K9B | 3.31783 | 32.92669 | 949 |  |  | √ |
| 15 | K10A | 3.27121 | 32.94735 | 980 |  |  | √ |
| 16 | K10B | 3.27121 | 32.94735 | 980 |  |  | √ |
| 17 | K11A | 3.33963 | 32.55141 | 980 |  |  | √ |
| 18 | K11B | 3.33963 | 32.55141 | 980 |  |  | √ |
| 19 | S10 | 1.68646 | 33.55708 | 1074 |  |  | √ |
| 20 | S13 | 1.71619 | 33.58256 | 1105 |  |  | √ |
| 21 | S25 | 1.71619 | 33.58256 | 1105 |  |  | √ |
| 22 | S31 | 1.70317 | 33.60989 | 1177 |  |  | √ |
| 23 | S4A | 1.79975 | 33.63371 | 1128 |  |  | √ |
| 24 | S5A | 1.80355 | 33.63127 | 1118 | √ | √ | √ |
| 25 | S6A | 1.8076 | 33.6342 | 1114 | √ |  | √ |
| 26 | S7A | 1.80565 | 33.6325 | 1121 |  | √ | √ |
| 27 | S8B | 1.8096 | 33.63093 | 1117 |  | √ | √ |
| 28 | S9A | 1.78872 | 33.64265 | 1129 | √ |  | √ |
| 29 | S13A | 1.79872 | 33.64265 | 1129 |  | √ | √ |
| 30 | S24A | 1.97259 | 33.70563 | 1108 | √ |  | √ |
| 31 | S25A | 1.99927 | 33.70563 | 1117 |  |  | √ |
| 32 | S26A | 1.0253 | 33.68600 | 1142 | √ | √ | √ |
| 33 | S27B | 1.92666 | 33.69059 | 1097 |  |  | √ |
| 34 | S30A | 1.92636 | 33.69735 | 1096 | √ |  | √ |
| 35 | S17A | 1.46264 | 33.26073 | 1064 |  |  | √ |
| 36 | S33B | 1.46264 | 33.26073 | 1064 | √ |  | √ |
| 37 | M1A | 3.17597 | 30.9572 | 1038 |  |  | √ |
| 38 | M2A | 3.14943 | 30.90651 | 1038 | √ | √ | √ |
| 39 | M3A | 3.16475 | 30.90273 | 1038 |  |  | √ |
| 40 | M4A | 3.16426 | 30.89927 | 1038 |  |  | √ |
| 41 | M5A | 3.17745 | 30.38275 | 1038 | √ |  | √ |
| 42 | M6A | 3.18073 | 30.94709 | 1038 | √ |  | √ |
| 43 | M7A | 3.17047 | 30.91887 | 1038 |  |  | √ |
| 44 | M8A | 3.17737 | 30.88242 | 1038 | √ | √ | √ |
| 45 | M9A | 3.18131 | 30.94712 | 1038 | √ |  | √ |
| 46 | M10A | 3.16196 | 30.8959 | 1038 |  |  | √ |
| 47 | M11A | 3.19118 | 30.84743 | 1038 | √ | √ | √ |
| 48 | T1A | 3.14344 | 30.98772 | 1093 |  |  | √ |
| 49 | T2A | 3.12774 | 30.99614 | 1094 |  |  | √ |
| 50 | T3A | 3.1428 | 30.97749 | 1150 | √ |  | √ |
| 51 | T4A | 3.16481 | 31.00599 | 1062 |  |  | √ |
| 52 | T5A | 3.19038 | 31.09744 | 959 |  | √ | √ |
| 53 | T6A | 3.17195 | 31.02748 | 987 | √ |  | √ |
| 54 | T7A | 3.15102 | 31.06148 | 902 |  | √ | √ |
